# Detecting compensatory movements of stroke survivors using pressure distribution data and machine learning algorithms

**DOI:** 10.1186/s12984-019-0609-6

**Published:** 2019-11-04

**Authors:** Siqi Cai, Guofeng Li, Xiaoya Zhang, Shuangyuan Huang, Haiqing Zheng, Ke Ma, Longhan Xie

**Affiliations:** 1 Shien-Ming Wu School of Intelligent Engineering, South China University of Technology, Guangzhou, 510640 China; 2 The Third Affiliated Hospital, Sun Yat-sen University, Guangzhou, 510630 China

**Keywords:** Stroke, Reaching, Machine learning, Classification, Pressure

## Abstract

**Background:**

Compensatory movements are commonly employed by stroke survivors during seated reaching and may have negative effects on their long-term recovery. Detecting compensation is useful for coaching the patient to reduce compensatory trunk movements and improving the motor function of the paretic arm. Sensor-based and camera-based systems have been developed to detect compensatory movements, but they still have some limitations, such as causing object obstructions, requiring complex setups and raising privacy concerns. To overcome these drawbacks, this paper proposes a compensatory movement detection system based on pressure distribution data and is unobtrusive, simple and practical. Machine learning algorithms were applied to classify compensatory movements automatically. Therefore, the purpose of this study was to develop and test a pressure distribution-based system for the automatic detection of compensation movements of stroke survivors using machine learning algorithms.

**Methods:**

Eight stroke survivors performed three types of reaching tasks (back-and-forth, side-to-side, and up-and-down reaching tasks) with both the healthy side and the affected side. The pressure distribution data were recorded, and five features were extracted for classification. The *k*-nearest neighbor (*k*-NN) and support vector machine (SVM) algorithms were applied to detect and categorize the compensatory movements. The surface electromyography (sEMG) signals of nine trunk muscles were acquired to provide a detailed description and explanation of compensatory movements.

**Results:**

Cross-validation yielded high classification accuracies (F1-score>0.95) for both the *k*-NN and SVM classifiers in detecting compensation movements during all the reaching tasks. In detail, an excellent performance was achieved in discriminating between compensation and noncompensation (NC) movements, with an average F1-score of 0.993. For the multiclass classification of compensatory movement patterns, an average F1-score of 0.981 was achieved in recognizing the NC, trunk lean-forward (TLF), trunk rotation (TR) and shoulder elevation (SE) movements.

**Conclusions:**

Good classification performance in detecting and categorizing compensatory movements validated the feasibility of the proposed pressure distribution-based system. Reliable classification accuracy achieved by the machine learning algorithms indicated the potential to monitor compensation movements automatically by using the pressure distribution-based system when stroke survivors perform seated reaching tasks.

## Background

Stroke is an important cause of death and the most frequent cause of adult-acquired disability, with up to 80% of stroke survivors suffering from upper-limb impairments that have a severe impact on the survivor’s ability to perform daily activities and influence their quality of life [1, 2]. Stroke survivors often compensate for the loss of motor function by adapting their movement patterns to incorporate additional degrees of freedom at other joints and body segments. During the seated reaching motion with their paretic upper limb, many patients spontaneously employed replace the use of their arm by recruiting excessive trunk or scapular movements, even though they are able to use their arm when forced to do so [3].

Although compensatory movements help patients obtain an immediate improvement of function, compensation may be detrimental to the final functional outcome of the impaired arm in the long-term [4, 5]. Specifically, the presence of excessive trunk movement in stroke survivors while reaching may lead to nonoptimal movement patterns, hindering further improvement and jeopardizing the potential recovery of their paretic upper limb [4, 5]. Moreover, there is also evidence that reducing compensatory trunk movements, for instance using a trunk restraint, may produce greater improvements in the upper limb impairment and function [6, 7] This highlights the need to monitor compensatory movements to optimize rehabilitation of stroke survivors.

Currently, the available approaches to detecting compensatory movements are based on sensor-based and camera-based detection systems. Sensor-based systems are commonly used to monitor the posture and upper limb movements of stroke patients in rehabilitation [8]. Accelerometers [9], inertial measurement units (IMU) [10], sensing garments [11] or other sensors are placed on the patients to monitor the trunk and/or shoulder compensatory movements. The main drawback of the sensor-based systems is the possibility of inducing unnatural movements due to the attached sensors. It is difficult to find an unobtrusive and easy-to-use solution [12]. Moreover, the validity and reliability of the outcome estimates from these wearable sensors for rehabilitation of stroke survivors is a daunting challenge for researchers [13, 14]. Other works on detecting compensatory movements of stroke survivors in rehabilitation have relied primarily on camera-based technology [15], including the marker-based and markerless human movement tracking technologies. The marker-based motion capture technologies can obtain more accurate and robust 3D tracking results but need expensive specialized hardware and require an elaborate setup [13, 16]. The marker-free methods enable simple, time-efficient, and potentially more meaningful assessments of human movement in clinical practice by eliminating the need for markers [17, 18]. However, the accuracy of markerless methods is still technically challenging [19]. Generally, camera-based technologies, both marker-based and markerless methods, are not appropriate for use in clinical settings since camera-based systems are not portable and require a space with a clear line-of-sight for the cameras and complex setups. Camera-based systems also introduce issues with respect to privacy and may cause unnatural behaviors due to the negative feelings caused by being monitored [20].

Given the limitations of sensor-based and camera-based systems, it is critical to develop a simple, unobtrusive, practical and low-cost method to detect compensatory movements of stroke survivors. We proposed a novel method for detection of compensatory movement patterns using the pressure distribution and machine learning algorithms. The pressure distribution of a person seated on a chair was assessed by a pressure distribution mattress, which consists of matrices of usually piezoresistive effect-based sensors [21, 22]. Considering that compensation movement mainly includes three types of movement patterns—trunk lean forward, trunk rotation and shoulder elevation, during seated reaching [23], the pressure distribution in the chairs has the potential to reflect these three compensatory movement patterns and can serve as a compensation detection method. To date, some investigators have adopted several machine learning algorithms for classifying the sitting postures based on the pressure distribution data and obtained a sufficient classification accuracy [22, 24–26]. To our knowledge, no previous study has evaluated the feasibility and validity of machine learning methods to detect compensatory movements based on the pressure distribution data of stroke survivors. Recently, we proposed and tested the use of a pressure distribution mattress to detect compensatory motions on healthy subjects [27]. Machine learning method was implemented to classify compensation.

based on pressure data and obtained good reliability and precision. However, real compensatory movements by stroke survivors is different from how healthy people simulate compensatory movements. To use this method in stroke survivors, the classifier needs to be adaptive to the variation in compensatory movements.

Therefore, the purpose of this study was to detect compensatory movements from the pressure distribution data of stroke survivors using machine learning methods. The *k*-nearest neighbor (*k*-NN) and support vector machine (SVM) methods were applied to classify the normal and compensatory movement patterns (trunk lean forward, trunk rotation and shoulder elevation) during three basic seated reaching tasks, including side-to-side, back-and-forth, and up-and-down reaching. Furthermore, the surface electromyography (sEMG) signals, which can reflect the degree of activity of the muscles [28], was used to provide a more detailed description of compensatory movements and verify the classification results.

## Methods

### Participants

Eight stroke survivors with varying degrees of upper-limb mobility impairment were recruited from the Third Affiliated Hospital, SUN Yat-sen University in this study (Table 1). They were recruited by therapist referral and through the central recruiting process at the hospital. All the participants provided informed consent, and the procedures were approved by the Guangzhou First People’s Hospital Department of Ethics Committee.

**Table 1:** Demographic and clinical characteristics (*N* = 8)

	Age	Sex	Affected Side	Weight (kg)	Month post stroke	UE-FMA^a^
P1	54	F	Left	60	2	34
P2	45	M	Left	54	3	55
P3	68	F	Left	39.5	2	38
P4	50	M	Right	78	13	48
P5	39	M	Left	80	8	38
P6	52	M	Left	65	6	19
P7	37	M	Right	72.5	5	32
P8	65	M	Right	65	9	35

^a^Upper extremity of the Fugl-Meyer Assessment (0–66 points)

Participants met the following criteria.

Inclusion criteria were as follows: 1) first ever stroke, 2) stroke survivor either in the subacute (between 1 to 6 months post stroke) or chronic (over 6 months post stroke) stage of recovery, 3) a fair to good cognitive level (Mini Mental State Examination (MMSE) score ≥ 24 [29]), 4) Ability to perform.

the required motions, and 5) Ability to remain in a sitting posture.

Exclusion criteria were as follows: 1) upper limb pain > 4/10 on a Visual Analogue Scale (VAS) [30], 2) upper limb spasticity > 2 on the Modified Ashworth Scale (MAS) [31], and 3) visual spatial neglect based on clinical judgment.

### Experimental setup and instruments

The pressure distribution data of the stroke survivors were recorded during the seated reaching tasks with a commercially available pressure distribution mattress (Body Pressure Measurement System (BPMS), Model 5330, Tekscan, Inc., South Boston, MA, USA). The sensor sheet is flexible (0.20 mm in thickness) and has a high resolution, with 1024 (32 × 32) piezoresistive pressure sensors covering approximately 471 mm × 471 mm of the total pressure sensitive area. The BPMS system can measure the body pressure distribution with minimal interference of the support surface, including the location of the pressure, magnitude of the peak pressures, and the overall pressure distribution patterns. The sensor is read sequentially by driving one of the rows and sensing one of the columns. The microprocessor selects the row and column to be read by identifying the proper address for each intersecting row and column. The pressure distribution data were recorded at a measurement frequency of 50 Hz.

**Fig. 1:**
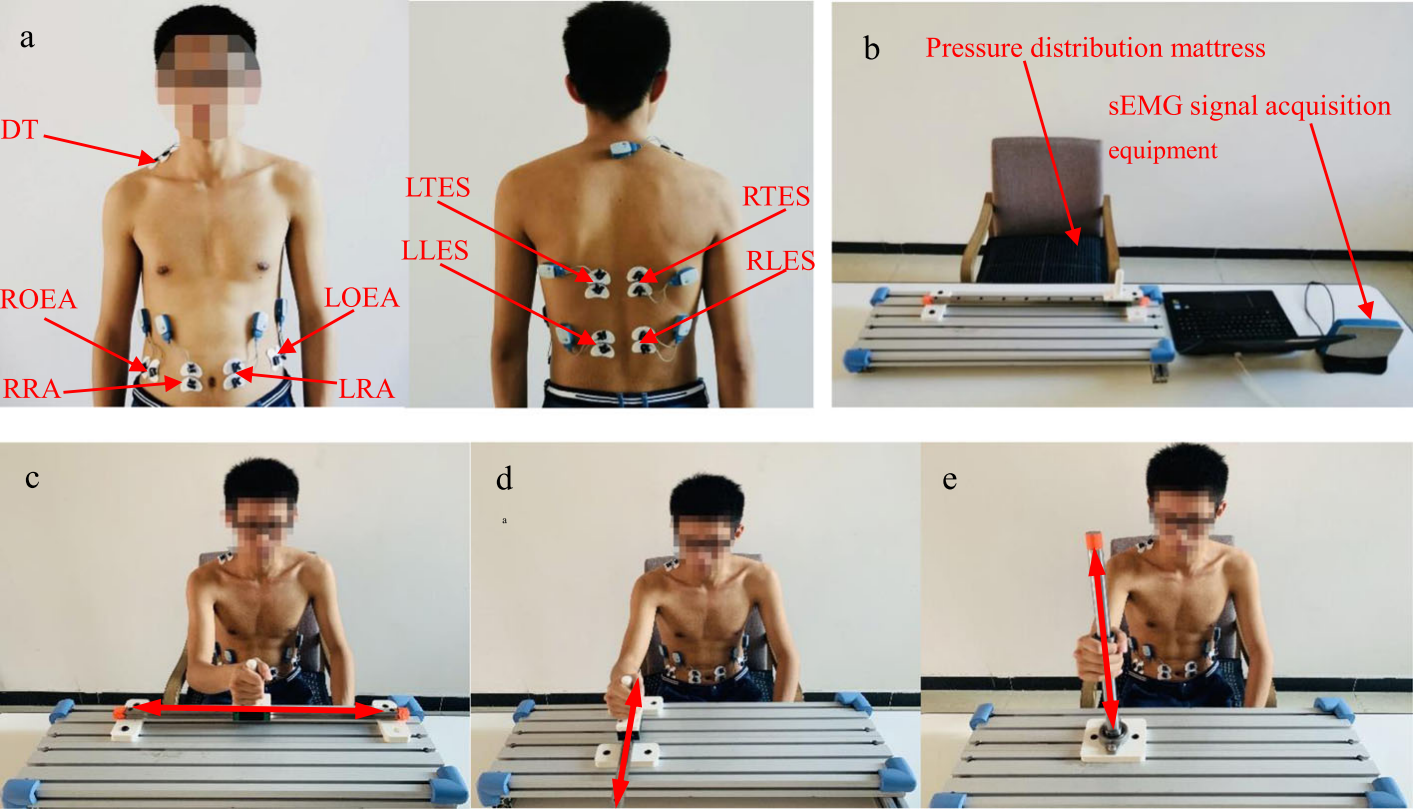
The experimental setup and three types of reaching tasks. **a** Electrode placement on the trunk muscles. **b** Experimental platform for seated reaching. **c** Side-to-side reaching. **d** Back-and-forth reaching. **e** Up-and-down reaching

In order to monitor the states of the trunk muscles during movements, 9 trunk muscles were selected, including the left and right rectus abdominis (LRA and RRA), left and right obliquus externus abdominis (LOEA and ROEA), left and right thoracic erector spinae (LTES and RTES), left and right lumbar erector spinae (LLES and RLES) and the descending part of the trapezius muscle (DT) corresponding to the moving side of the upper limb. The sEMG signals of the 9 muscles were recorded using bipolar surface Ag/AgCl electrodes (Pirronse & Co., Italy) attached approximately 2 cm apart along the longitudinal axis of the muscle belly, as shown in Fig. 1a. The collection of the sEMG signals was strictly in accordance with the recommended standards [32].

The participants sat comfortably in front of a table on an adjustable chair with a back support that did not restrict the trunk movements. The pressure distribution mattress was mounted on the chair. The height of the chair was adjusted to the length of the legs of the participants so that their feet were flat on the floor. Each participant performed the three basic reaching tasks that were selected to cover a wide range of movements of the arm at the shoulder and elbow. These tasks were (i) side-to-side reaching (Fig. 1c), (ii) back-and-forth reaching (Fig. 1d), and (iii) up-and-down reaching (Fig. 1e). During the reaching motions, three types of compensatory synergies were commonly elicited, including an excessive axial trunk rotation (TR), trunk lean-forward (TLF) [33, 34] and shoulder elevation movements (SE) [13, 15].

Each subject performed the three types of reaching tasks with his/her affected side and healthy side. Each reaching task was repeated 30 times at a self-selected speed. Thus, each subject performed 180 motions totally, with 90 motions for each side. Motions performed by the subjects’ healthy side were labeled as NC movements. To avoid fatigue, the subjects were allowed 10 s of rest between two reaching tasks and 3 min of rest after a certain type of reaching task. The raw data of all the participants were recorded for the training and testing of the pressure sensor-based detection method of compensation in upper-limb movements.

### Data processing

All the sEMG recordings were digitized at 2 kHz using the myoMUSCLE (NORAXON, Arizona) and were processed using MATLAB software (MathWorks Corp., Natick, MA, USA). Data preprocessing methods, including baseline correction, a 20–200 Hz bandpass filter, power frequency filter (50 Hz notch), full-wave rectification, and amplitude normalization, were carried out to improve the signal-to-noise ratio of the sEMG signals [35]. All the filters used in this paper are 4th-order Butterworth filters [36]. The root mean square (RMS) was calculated to evaluate the level of activity of the trunk muscles [37]. The average values of the RMS_sEMG_ across the 8 patients were calculated as Ave-RMS_sEMG_.

**Fig. 2:**
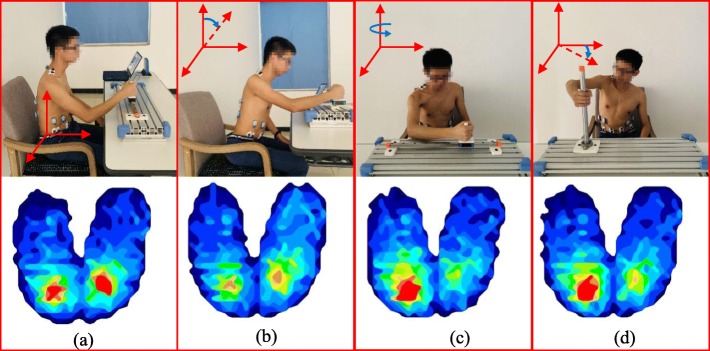
The pressure map of the four postures. **a**) Sitting straight; **b**) Sitting with the trunk leaning forward; **c**) Sitting with trunk rotation; **d**) Sitting with shoulder elevation

The pressure distribution data of the 1440 motions (180 × 8) were acquired by using the BPMS software and exported into the ASCII format for postprocessing in MATLAB. Each pressure map consists of a 32 × 32-dimensional vector, and the pressure sensor values were preprocessed before extracting the features. Since the pressure mattress modules have a unique default offset level, a bias value matrix was recorded on a regular basis and used for offset data removal. The pressure maps of the four typical postures, including sitting up straight, sitting with the trunk leaning forward, sitting with trunk rotation and sitting with shoulder elevation, were displayed as a color-coded real-time display (Fig. 2).

### Feature extraction

The pressure sensor array is represented as a set of indexed sensors {*P*_1_[*t*], *P*_2_[*t*], ⋯, *P*_*N*_[*t*]] }, where *N* = 1024 is the total number of sensors in the array. Each sensor is represented as a triple, *P*_*i*_[*t*] = (*x*_*i*_, *y*_*i*_, *p*_*i*_(*t*)), where *x*_*i*_ and *y*_*i*_ are the lateral and longitudinal coordinates of the *ith* sensor, respectively, and *p*_*i*_(*t*) is the sensor value at time *t*.

By reviewing these pressure distribution data and existing research of pressure distribution mattresses [22, 38], five features were extracted for classification, including the average sensor value (ASV), standard deviation of the lateral center of pressure (SD_LatCOP_), standard deviation the of longitudinal center of pressure (SD_LonCOP_), the standard deviation of the ratio of the left-side to right-side pressure (SD_LRratio_) and the standard deviation of the ratio of front-side to back-side pressure (SD_FBratio_).

The ASV was used to reflect the average amplitude of the pressure data in each reaching task.

1 $$ ASV= SSV/T $$

where T is the total time and *SSV* is the sum of the pressure sensor values ,*SSV*(*t*). *SSV*(*t*) was obtained from the sum of *p*_*i*_(*t*) for all *i* at a given time *t*.

2 $$ SSV(t)=\sum \limits_{i=1}^N{p}_i(t) $$

3 $$ SSV=\sum \limits_{t=1}^T SSV(t) $$

SD_LatCOP_, SD_LonCOP_, SD_LRratio_ and SD_FBratio_ are the standard deviations of the lateral center of pressure (LatCOP), longitudinal center of pressure (LonCOP), ratio of the left-side to right-side pressure (LRratio) and ratio of the front-side to back-side pressure (FBratio), respectively, which were used to reflect the volatility of the different reaching tasks.

4 $$ LatC\mathrm{O}P(t)={\sum}_{i=1}^N{x}_i{p}_i(t)/ SSV(t) $$

5 $$ LonCOP(t)={\sum}_{i=1}^N{y}_i{p}_i(t)/ SSV(t) $$

6 $$ LRratio(t)={\sum}_{y_i=1}^{y_i=16}{p}_i(t)/{\sum}_{y_i=17}^{y_i=32}{p}_i(t) $$

7 $$ FBratio(t)={\sum}_{x_i=1}^{x_i=16}{p}_i(t)/{\sum}_{x_i=17}^{x_i=32}{p}_i(t) $$

### Classification

Based on our work on healthy subjects [27], The *k*-NN classifier [39] and SVM [40, 41] were used to classify the normal and compensatory movement patterns of the participants.

The *k*-NN classification algorithm is a nonparametric classification method, which is simple but effective in many cases [41]. The *k*-NN algorithm works by using an input vector with the *k* closest training samples in the feature space. The classification of the data was performed by identifying the most common class among the *k* nearest neighbors. The algorithm requires training to define the neighbors based on the distance from the test sample and a testing step to determine the class to which this test sample belongs. In this study, the distance is measured using the Euclidean distance. The Euclidean distance between *n* dimensional attribute vectors *X* = (*x*_1_, *x*_2_, …, *x*_*n*_) and *Y* = (*y*_1_, *y*_2_, …, *y*_*n*_) can be defined by eq. (8). To determine the k value, the *k*-NN algorithm was run with different *k* values, and the one with the best performance was chosen.

8 $$ dist\left(X,Y\right)=\sqrt{\sum \limits_{i=1}^n{\left({x}_i-{y}_i\right)}^2} $$

The SVM algorithm performs classification tasks by mapping the features onto a multidimensional space and constructing the decision boundaries, called hyperplanes, which maximize the margin between the observations of different activity classes. The margin is determined by the distance between the “support vectors”, which are the observations that lie in an area of space that creates a boundary between the activity classes [42]. The principle of segmentation is to maximize the interval and finally transform it into a convex quadratic programming problem to solve [43], expressed as:

9 $$ \left\{\begin{array}{c}\mathit{\min}\frac{1}{2}{\left\Vert \boldsymbol{w}\right\Vert}^2\\ {}s.t.{y}_i\left({\boldsymbol{w}}^T{\boldsymbol{x}}_i+b\right)-1\ge 0,i=1,2,..,N\ \end{array}\right. $$

where (***x***_*i*_, *y*_*i*_) is the *t* th data point and (***w***, *b*) is the hyperplane parameter. The Lagrange multiplier technique is applied to solve the problem in this study.

For the implementation of the SVM models, the features were normalized to a mean of zero and scaled to unit variance by subtracting the corresponding mean and dividing by the standard deviation. Since the SVM algorithm only considers the samples close to the class boundary, it shows higher stability in small training sets, even when high dimensional data sets are classified [44].

The extracted features, including the ASV, SD_LatCOP_, SD_LonCOP_, SD_LRratio_ and SD_FBratio_, were supplied to the *k*-NN and SVM classifiers. Four-fold cross-validation was used to assess classifier performance [45–47]. Six subjects were randomly selected to form the training data sets, while data of the other two subjects formed the testing data sets. The process is repeated until the data for each subject is used as a test dataset, and the results are aggregated to verify the models and find the average recognition rate of the system. The confusion matrix displays the relationship between the observed and predicted values obtained by a classifier and is a convenient tool for evaluating the classification performance [48]. Based on the confusion matrix, three accuracy metrics, precision, recall and F1-score, can be obtained. Precision describes the accuracy of the detection, while recall describes how well the target objects are detected without being missed. The F1-score combines the precision and recall metrics and is minimally biased by class size imbalances [49].

### Statistical analysis

Statistical analysis was performed using IBM SPSS statistics software (ver. 24.0, IBM Corp., Armonk, NY, USA)). Differences in the F1-scores of the *k*-NN and SVM classifiers were tested for statistical significance using a paired t-test. The differences in the F1-scores across eight stroke survivors were tested for statistical significance using the Friedman nonparametric tests. A paired t-test was used to compare the Ave-RMS_sEMG_ of LRA, RRA, LOEA, ROEA, LTES, RTES, LTES, RLES and DT between the healthy side and the affected side across eight subjects. In addition, a paired t-test was used to compare the Ave-RMS_sEMG_ of the healthy side and the affected side. A significance level of *P* < 0.05 was used for all the analyses.

## Results

The classification performance of the *k*-NN and SVM algorithms in detecting compensation movements during three types of reaching tasks, including side-to-side reaching, back-and-forth reaching, and up-and-down reaching, is shown in Table 2. The results showed that generally good classification performance was achieved, with all the F1-scores above 0.95. The best performance for recognizing compensation movements was obtained for the up-and-down reaching, with a high F1-score (0.998) of both the *k*-NN and SVM classifiers. The SVM classifier detected compensation movement for back-and-forth reaching with a higher F1-score (0.994) than the *k*-NN classifier (F1-score = 0.992), while the *k*-NN classifier detected compensation movement for side-to-side reaching with a higher F1-score (0.990) than the SVM classifier (F1-score = 0.987). An average F1-score of 0.993 was achieved by both the *k*-NN classifier and SVM classifier, which demonstrated the excellent classification performance.

**Table 2:** Classification performance of the *k*-NN and SVM classifiers in detecting compensation movements

		Back-and-forth reaching	Side-to-side reaching	Up-and-down reaching	Average
*k*-NN	Precision	0.984	0.988	0.996	0.989
	Recall	1.000	0.992	1.000	0.997
	F1-score	0.992	0.990	0.998	0.993
SVM	Precision	0.988	0.996	0.996	0.993
	Recall	1.000	0.979	1.000	0.993
	F1-score	0.994	0.987	0.998	0.993

*k*-NN = *k*-nearest neighbor, SVM = support vector machine

**Fig. 3:**
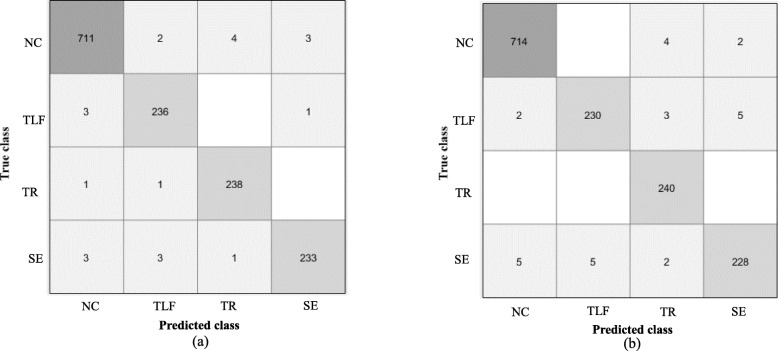
A representative confusion matrix of one classification for the four motions. **a**) *k*-NN classifier with an accuracy of 0.985. **b**) SVM classifier with an accuracy of 0.981. NC = Noncompensation, TLF = Trunk lean-forward, TR = Trunk rotation, SE = Shoulder elevation, *k*-NN = *k*-nearest neighbor, SVM = support vector machine

The *k*-NN and SVM algorithms were used to classify four different motions, including three types of compensatory motions (TLF, TR and SE) and NC motion. The confusion matrix of one classification for the four motions with an accuracy of 0.985 achieved by the *k* -NN classifier is shown in Fig. 3a, and an accuracy of 0.981 achieved by the SVM classifier is shown in Fig. 3b.

The precision, recall and F1-score of each classification algorithm were calculated to evaluate the performance, as shown in Table 3. Both the *k*-NN and SVM classifiers exhibited good performances in the multiclass classification with an average F1-score of 0.981. The SVM classifier detected the NC motion with an excellent accuracy (F1-score = 0.990), followed by TR (F1-score = 0.983), TLF (F1-score = 0.975) and SE (F1-score = 0.975). Compared with the SVM classifier, the *k*-NN classifier achieved a higher F1-score in detecting SE (0.981) and a lower F1-score in detecting TLF (0.970).

**Table 3:** Classification performance-three types of compensatory motions

		NC	TLF	TR	SE	Average
*k*-NN	Precision	0.986	0.987	0.979	0.979	0.983
	Recall	0.993	0.954	0.988	0.983	0.980
	F1-score	0.989	0.970	0.983	0.981	0.981
SVM	Precision	0.989	0.996	0.975	0.967	0.982
	Recall	0.992	0.954	0.992	0.983	0.980
	F1-score	0.990	0.975	0.983	0.975	0.981

*NC* = Noncompensation, *TLF* = Trunk lean-forward, *TR* = Trunk rotation, *SE* = Shoulder elevation, *k*-NN = *k*-nearest neighbor, *SVM* = support vector machine

**Fig. 4:**
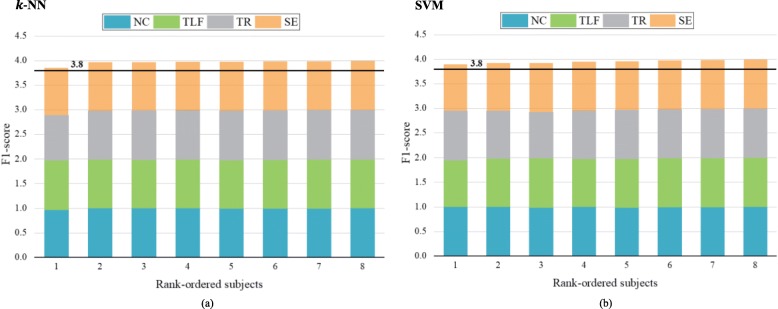
Stacked bar graph of the F1-scores for the *k*-NN and SVM classifiers for all the classes and participants. The participants rank ordered based on the total value of the F1-score in the presented classes. The black horizontal line represents a general cutoff for the highly functional levels of classification performance (average F1-score > 0.95). **a**) Stacked bar graph of F1-scores from the k-NN classifier. **b**) Stacked bar graph of F1-scores from the SVM classifier. NC = Noncompensation, TLF = Trunk lean-forward, TR = Trunk rotation, SE = Shoulder elevation, *k*-NN = *k*-nearest neighbor, SVM = support vector machine

Figure 4 shows how each class performs using the *k*-NN and SVM classifiers across all participants (*N* = 8). Good classification performance was achieved by both the *k*-NN (average F1-score = 0.991 ± 0.016) and SVM (average F1-score = 0.987 ± 0.017) algorithms for all the classes and participants. In terms of the *k*-NN classifier, the minimum and maximum F1-scores of the four classes in all subjects were 1.000 and 0.915, respectively. In terms of the SVM classifier, the minimum and maximum F1-scores of the four classes in all the subjects were 1.000 and 0.933, respectively. The F1- scores across the eight participants were not significantly different between the *k*-NN and SVM classifiers (paired *t*-test: *P* = 0.362). Though eight survivors with different level of severity, the Friedman nonparametric test indicated that differences in the F1-scores across the eight participants were not statistically significant (*k*-NN classifier, *p* = 0.653; SVM classifier, *p* = 0.715).

### Relationship with muscle activity

**Fig. 5:**
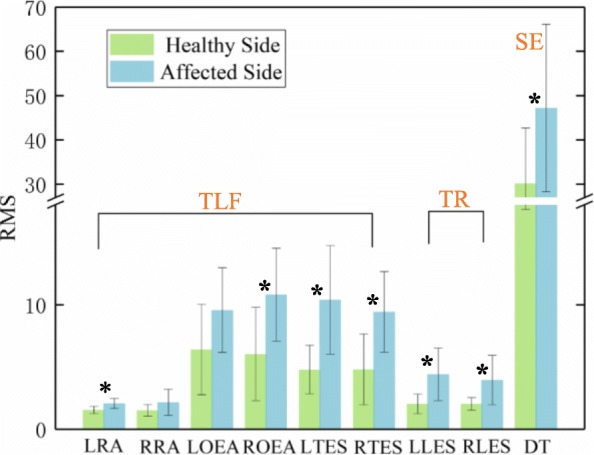
Ave-RMS_sEMG_ of the 9 trunk muscles, including the LRA, RRA, LOEA, ROEA, LTES, RTES, LTES, RLES and DT, during the TLF, TR and SE movements using the healthy side and the affected side. * indicates significant difference (paired t-test, *p* < 0.05) between the healthy side and the affected side across eight subjects. TLF = Trunk lean-forward, TR = Trunk rotation, SE = Shoulder elevation

The relationship between muscle activity and compensatory patterns was analyzed based on the sEMG signals using the RMS features. The RMS_sEMG_ of the 9 trunk muscles of each patient, including the LRA, RRA, LOEA, ROEA, LTES, RTES, LTES, RLES and DT (Fig. 1a), were calculated. The average values of the RMS_sEMG_ across the 8 patients were obtained as Ave-RMS_sEMG_, and the paired *t*-test (*P* = 0.027) provided evidence of a statistically significant difference between the healthy side and the affected side, as shown in Fig. 5. Trunk compensatory patterns reflected by sEMG signals were consistent with recognized based seat pressure distribution data. In general, the Ave-RMS_sEMG_ of the LRA, RRA, LOEA, ROEA, LTES and RTES of the healthy side was smaller than those of the affected side, which indicated the TLF movement pattern. The Ave-RMS_sEMG_ of LLES and RLES of the healthy side was smaller than those of the affected side, which indicated the TR movement pattern. The Ave-RMS_sEMG_ of DT of the healthy side was smaller than those of the affected side, which indicated the SE movement pattern. The high standard deviation associated with these compensatory movements underscored the great variability in the trunk muscle activity required by the different patients.

## Discussion

This is the first time that detecting compensation movements during seated reaching using machine learning algorithms from the pressure distribution data of stroke survivors has been studied. Our classifiers were trained on the features from the pressure distribution data, including the ASV, SD_LatCOP_, SD_LonCOP_, SD_LRratio_ and SD_FBratio_, and achieved excellent classification performances in discriminating between compensation and noncompensation movements during three types of reaching tasks that are routinely performed by patients with stroke. Furthermore, our classifiers exhibited good performance in recognizing three types of compensatory strategies, including TLF, TR and SE, which provides important feedback for both patients and clinicians and contributes to reducing compensation accordingly.

By using the *k*-NN and SVM classifiers, the pressure distribution-based system displayed comparable classification performance with sensor-based and camera-based systems. Ranganathan R et al. [20] used a sensor-based system to detect the compensation and noncompensation movements during reaching tasks in stroke survivors. An average F1-score of 0.890 was obtained for side-and-side reaching, while a higher average F1-score of 0.962 was obtained for up-and-down reaching. Our methods showed better classification performance, with an average F1-score of 0.989 for side-and-side reaching and 0.998 for up-and-down reaching. Babak Taati et al. [12, 16, 23] applied a camera-based system to identify and categorize compensatory movements by a multiclass classifier. An acceptable accuracy (86% per frame) was achieved in healthy adult subjects who were asked to simulate a series of compensation movements. However, the detection accuracy was worse in stroke survivors for TLF compensation (F1-score = 0.17), TR compensation (F1-score = 0.27) and SE compensation (F1-score = 0.07). The classification performance indicated that the motion captured from the stroke survivors could not be used to train an accurate posture detection classifier. In contrast, our classifiers exhibited good classification performances in the multiclass classification of compensatory movements, with an average F1-score of 0.973 for TLF compensation, 0.983 for TR compensation and 0.978 for SE compensation. These results validated that the pressure distribution-based system can adequately detect and categorize compensatory movements during seated reaching in patients after a stroke.

Existing methods for detecting compensatory movements, both sensor-based and camera-based systems, mainly focus on the kinematic parameters of the participants, such as the movement angle, distance, speed and acceleration. The precision of the kinematic parameters is affected easily, and it may also explain why the performance of the classifiers based on the kinematic parameters individually was not reliable enough to discern the compensation movements in stroke survivors during seated reaching. Considering that the pressure distribution data of stroke survivor’s body is much larger than the pressure caused by other sensors attached to his/her body, the classification performance of detecting compensations can remain reliable and stable even if there are additional sensors. Thus, combining the different sensor data, such as the pressure distribution data, may be a suitable solution to improve the accuracy of detection of compensatory movements. sEMG signals also showed the potential to reflect compensatory movements of stroke patients, although the muscle activity of different patients has great variability. Research on a larger sample of stroke survivors at different phases of recovery and with different levels of upper limb impairment is recommended.

This study had a number of strengths. First, we validated the feasibility of detecting compensatory movements of stroke survivors during seated reaching using pressure distribution data. Second, machine learning algorithms were implemented to detect and categorize compensatory movements and achieved excellent classification performances. Third, the pressure distribution-based system is more practical and cost-effective in clinical and home settings, since it does not suffer from object obstruction and complex setup. Finally, detecting compensatory motions based on pressure distribution data could help to monitor the patient’s postures and movements without the need for direct supervision by a therapist. Additional information, for instance, how many times the patient compensated during a rehabilitation training and which kind of compensatory patterns the patient employed, can be obtained by our compensatory movement detection system. These new information about motor compensations may help to understand and access the underlying motor deficits in patients with stroke.

One limitation of this study is that we detected and categorized compensatory movements instead of assessing compensation quantitatively. Our classifiers can detect the presence or absence of compensatory movements accurately and reliably. Though eight survivors with different level of severity, the Friedman nonparametric test indicated that differences in the F1-scores across the eight participants were not statistically significant (*k*-NN classifier, *p* = 0.653; SVM classifier, *p* = 0.715). But these classifiers cannot provide additional information about about the levels of compensation. Therefore, we only can analyze the relation between the sEMG and seat pressure distribution data qualitatively instead of quantitatively. From a clinical perspective, distinguishing different levels of compensatory trunk movement, such as mild, moderate and severe, may have the potential to provide a more detailed description of compensatory movements and guide rehabilitation more reasonably. Meanwhile, considering that compensatory patterns are more variable in stroke patients with different levels of upper-limb impairment, it is possible that two or more compensatory movements are employed by stroke patients simultaneously. Since motions were labeled by the dominant compensatory pattern in this study, the classifier will detect the main compensation when multiple compensation occur simultaneously. Detecting compound compensation may provide more information to both the patients and therapists.

### Future work

Consistent with the results of previous studies [50–52], stroke survivors exhibited significantly higher trunk movements during seated reaching tasks with their affected arm than with the healthy arm. Excessive use of compensatory movements can result in muscle contractures, joint misalignment, pain, limb disuse, and increased energy expenditure. After detecting the compensation movement, an appropriate trunk restraint technique should be incorporated to reduce the compensatory trunk movements during the seated reaching tasks. Visual, auditory or haptic feedback was provided to stroke survivors to modify their movement patterns and demonstrate the potential effects of improving upper limb function [53–55].

An accurate detection of compensatory movements was achieved using a pressure distribution-based system. In follow-up studies, we will develop an effective feedback system for stroke survivors based on the detection system and conduct clinical trials to test its feasibility and practicality.

## Conclusion

In summary, we have demonstrated the possibility of detecting compensation movements using machine learning classifiers from the pressure distribution data of stroke survivors. The *k*-NN and SVM algorithms consistently exhibited excellent classification performance in detecting and categorizing compensatory movements for three types of reaching tasks for all eight participants in this study. An average F1-score of 0.993 was obtained in discriminating between compensation and NC movements, and an average F1-score of 0.981 was achieved in recognizing NC, TLF, TR and SE. The accurate classification of the compensatory movements based on the use of pressure distribution data offers the potential to provide stroke survivors with precise and reliable feedback. Since the pressure distribution-based system is not limited to laboratory settings, it can be utilized to monitor compensatory movements during home training sessions. Future work will focus on developing an effective feedback system for stroke survivors based on the detection system to correct excessive compensatory trunk movements during seated reaching tasks as a potential supervision method.

## Data Availability

The datasets used and/or analyzed during the current study are available from the corresponding author upon reasonable request.

## Abbreviations

ASV: Average sensor value
BPMS: Body pressure measurement system
DT: Descending part of the trapezius muscle
FB ratio: Ratio of the front-side to back-side pressure
IMU: Inertial measurement units
LatCOP: Lateral center of pressure
LLES: Left lumbar erector spinae
LOEA: Left obliquus externus abdominis
LonCOP: Longitudinal center of pressure
LRA: Left rectus abdominis
LRratio: Ratio of the left-side to right-side pressure
LTES: Left thoracic erector spinae
MAS: Modified Ashworth Scale
MMSE: Mini mental state examination
NC: Noncompensation
RLES: Right lumbar erector spinae
RMS: Root mean square
ROEA: Right obliquus externus abdominis
RRA: Right rectus abdominis
RTES: Right thoracic erector spinae
*k*-NN: *k*-nearest neighbor
SE: Shoulder elevation
sEMG: Surface electromyography
SVM: Support vector machine
TLF: Trunk lean-forward
TR: Trunk rotation
UE-FMA: Upper extremity of the Fugl-Meyer Assessment
VAS: Visual Analogue Scale
